# *N*‐acyl homoserine lactone cell–cell diffusible signalling in the *Ralstonia solanacearum* species complex

**DOI:** 10.1111/mpp.13467

**Published:** 2024-08-04

**Authors:** Peng Li, Cristina Bez, Yong Zhang, Yinyue Deng, Vittorio Venturi

**Affiliations:** ^1^ Ministry of Education Key Laboratory for Ecology of Tropical Islands, Key Laboratory of Tropical Animal and Plant Ecology of Hainan Province, College of Life Sciences Hainan Normal University Haikou China; ^2^ International Centre for Genetic Engineering and Biotechnology Trieste Italy; ^3^ Interdisciplinary Research Center for Agriculture Green Development in Yangtze River Basin Southwest University Chongqing China; ^4^ School of Pharmaceutical Sciences (Shenzhen) Shenzhen Campus of Sun Yat‐sen University, Sun Yatsen University Shenzhen China; ^5^ African Genome Center, University Mohammed VI Polytechnic (UM6P) Ben Guerir Morocco

**Keywords:** AHL, LuxI/R, quorum sensing, *Ralstonia solanacearum*, regulatory network, RSSC

## Abstract

*Ralstonia solanacearum* species complex (RSSC) includes soilborne bacterial plant pathogens with worldwide distribution and wide host ranges. Virulence factors are regulated via four hierarchically organized cell–cell contact independent quorum‐sensing (QS) signalling systems: the Phc, which uses as signals (*R*)‐methyl 3‐hydroxypalmitate [(*R*)‐3‐OH PAME] or (*R*)‐methyl 3‐hydroxymyristate [(*R*)‐3‐OH MAME], the *N*‐acyl homoserine lactone (AHL)‐dependent RasI/R and SolI/R systems, and the recently identified anthranilic acid‐dependent system. The unique Phc QS system has been extensively studied; however, the role of the two AHL QS systems has only recently been addressed. In this microreview, we present and discuss current data of the SolI/R and RasI/R QS systems in the RSSC. We also present the distribution and frequency of these AHL QS systems in the RSSC, discuss possible ecological roles and evolutive implications. The complex QS hierarchical networks emphasizes the crucial role of cell–cell signalling in the virulence of the RSSC.

## Graphical abstract

Pathogens of the *Ralstonia solanacearum* species complex (RSSC) regulate virulence gene expression via four interconnected quorum‐sensing (QS) signalling systems ensuring a timely response in different environmental settings.

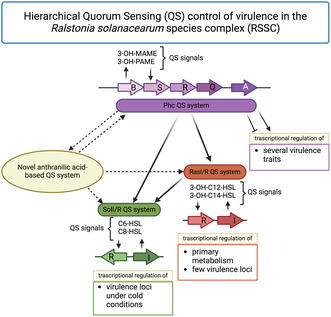

## 1 INTRODUCTION

Lethal wilts caused by members of the *Ralstonia solanacearum* species complex (RSSC) represent one of the most important bacterial diseases of plants (Fegan & Prior, 2005). The RSSC displays an unusual wide host range comprising over 200 plant species belonging to 50 different botanical families and these pathogens have been reported in more than 100 countries (Denny, 2006). Based on the phylogenetic clades, the RSSC is divided into phylotypes I, II, III, and IV. Recently, taxonomists suggested that the RSSC should be divided into three subgroups: *R. solanacearum* (phylotype II, Americas), *Ralstonia syzygii* (phylotype IV, Indonesia and Japan) and *Ralstonia pseudosolanacearum* (phylotype I, Asia; phylotype III, Africa; Prior et al., 2016; Safni et al., 2014).

As a soilborne and vascular pathogen, RSSC modulates virulence determinants during pathogenesis via an intricate regulatory network. At the initial infection stage, chemotaxis and flagellar motility contribute to sense and move towards root exudates and then the adhesin proteins allow attachment to the plant roots (Carter et al., 2023; Hayashi et al., 2019). After entering the host through wounds, RSSC undergoes extensive multiplication within the host, infecting the cortex and the xylem. This process involves the secretion of cell wall‐degrading enzymes and exopolysaccharides (EPS), leading to the inhibition of water flow and eventual cell death (Lowe‐Power et al., 2018; Peyraud et al., 2016). Other virulence determinants also contribute to the pathogenicity of RSSC as recently reviewed (Vailleau & Genin, 2023), including biofilm production (Mori et al., 2016), ralfuranones (Mori et al., 2018), type III secretion system effectors (T3Es; Landry et al., 2020), nitrate assimilation (Dalsing & Allen, 2014) and nitric oxide detoxification systems (Truchon et al., 2022). The regulation of virulence factor production, host colonization and infection process in the RSSC is heavily dependent on cell–cell quorum‐sensing (QS) signalling (Vailleau & Genin, 2023).

QS is a contact‐independent cell–cell signalling system involved in the regulation of virulence factors in many phytopathogens. QS systems consist of a signalling module that produces the signal and of a sensing module that interacts and responds to the signal, affecting the expression of target genes. These are involved in regulating a variety of bacterial community phenotypes in many different bacterial species, such as biofilm formation, toxin production, EPS synthesis, extracellular enzyme production, motility, and plasmid conjugation among many others. Several types of QS signals have been identified, mainly being either small organic molecules (smaller than 1000 Da) or peptides consisting of 5–20 amino acids. *N*‐acyl‐homoserine lactones (AHLs) are the most common signals produced by *Proteobacteria* including phytopathogens such as *Pantoea stewartii*, *Erwinia carotovorum*, *Pseudomonas syringae*, *Dickeya solani* and *Agrobacterium tumefaciens* where they play a key role in regulating virulence‐associated loci (Baltenneck et al., 2021; Papenfort & Bassler, 2016; Von Bodman et al., 2003). For example, in *P*. *stewartii*, the causal agent of Stewart's vascular wilt in maize, once bacterial colonization occurs, QS regulation at high cell density results in obstruction of water transport in the xylem by modulating biofilm formation (Papenfort & Bassler, 2016). Another well‐studied AHL QS system belongs to *A. tumefaciens* where it regulates the conjugative transfer and replication of the Ti plasmid (Fuqua & Winans, 1994). Another common signal is the diffusible signal factor (DSF) that is produced for example by *Xanthomonas* spp. (such as *X. oryzae* pv. *oryzae*, *X. campestris*) and *Xylella fastidiosa* phytopathogenic bacteria (He et al., 2023). DSF signals are *cis*‐2‐unsaturated fatty acids that can vary in carbon chain lengths (range from 8 to 14 carbons), double‐bond configurations, and side‐chain modifications, particularly methylation. In *Xanthomonas* spp. DSF synchronizes the expression of genes involved in the production of extracellular enzymes, biofilm formation, iron uptake and resistance to toxins and oxidative stress. Interestingly, it has also been shown that DSF is implicated in the intricate crosstalk between *Xanthomonas* spp. and their host plants, facilitating pathogen entry into the host and eliciting the plant innate immunity (Kakkar et al., 2015). Another QS system has been identified as highly conserved only among *Dickeya* spp. and is called VFM (virulence factor modulating). The *vfm* locus encodes a two‐component regulatory system VfmI/VfmH that is involved in the biosynthesis and export of the signal. Though the structure of the signal is currently unknown, it is involved in the regulation of the virulence and pathogenicity in several species of *Dickeya* spp. (Hugouvieux‐Cotte‐Pattat et al., 2022).

Bacterial strains belonging to the RSSC have evolved a unique QS system called Phc, which uses two different DSF‐derived signals called (*R*)‐methyl 3‐hydroxypalmitate [(*R*)‐3‐OH PAME] or (*R*)‐methyl 3‐hydroxymyristate [(*R*)‐3‐OH MAME]. The Phc QS system regulates EPS production, cellulolytic and pectolytic enzymes, motility as well as many other virulence‐associated factors (Flavier et al., 1997; Kai, 2023; Kai et al., 2015). The Phc system also regulates an AHL‐based QS system called SolI/R (Brumbley et al., 1993; Clough et al., 1997; Genin et al., 2005). Recently, another AHL‐based QS system called RasI/R was discovered in phylotype I, III and IV strains (Yan et al., 2022).

In this microreview, we aim to explore and discuss our current knowledge of the SolI/R and RasI/R AHL QS systems in RSSC, emphasizing their integration into an unique complex regulatory network that governs genes related to pathogenicity and host colonization. This microreview also provides an all‐inclusive picture of the distribution and frequency of the AHL QS systems in the RSSC complex, discussing their possible ecological role and evolutive advantage.

## 2 Phc QS IS THE CENTRAL REGULATORY SYSTEM CONTROLLING VIRULENCE IN THE RSSC

**FIGURE 1:**
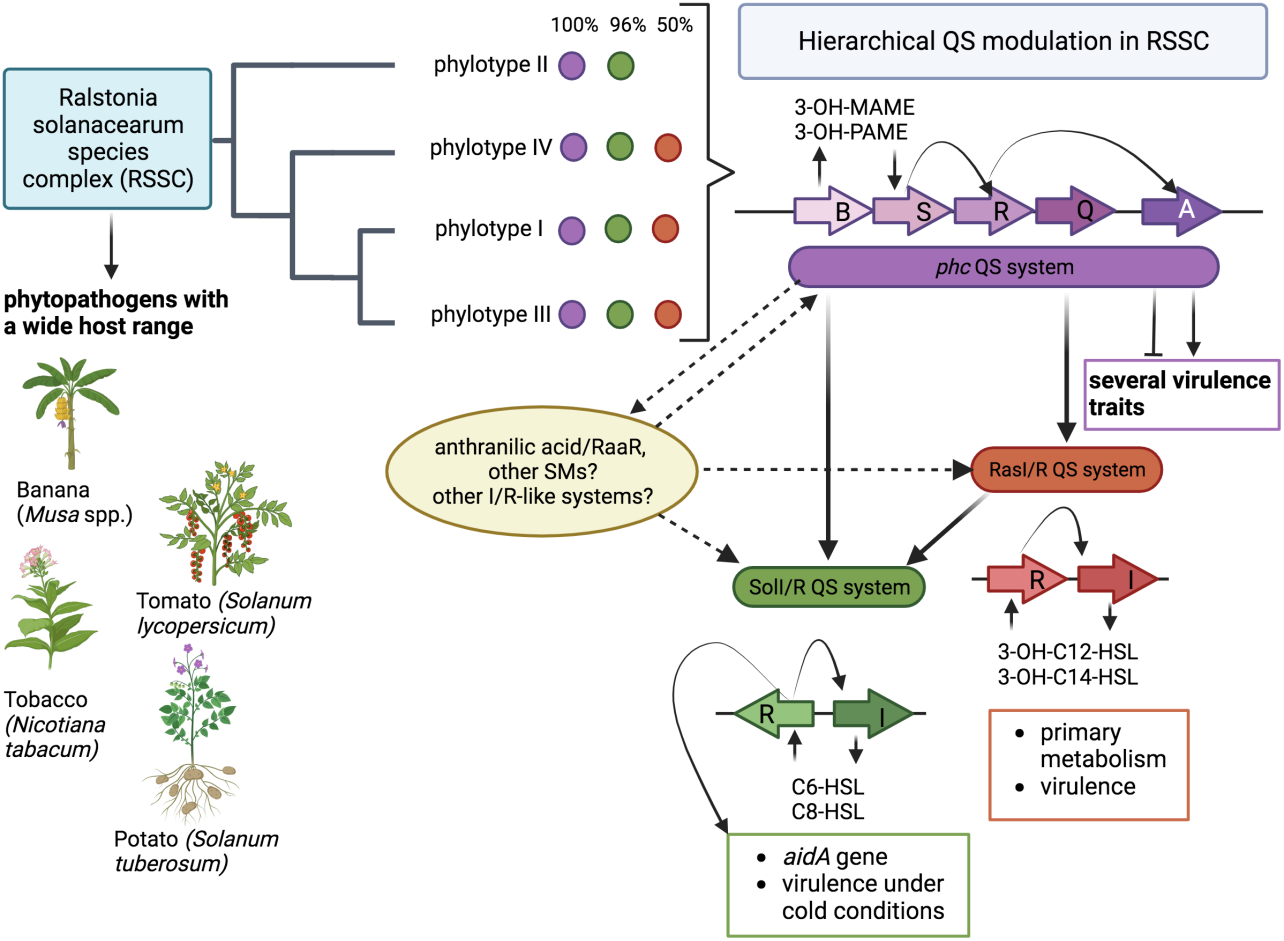
Schematic model illustrating the hierarchical quorum‐sensing (QS) modulation network as well as their phylogenetic and genetic organization in the *Ralstonia solanacearum* species complex (RSSC). The Phc QS system is depicted as purple and is harboured by all the RSSC strains (100%); SolI/R QS system is depicted as green and is found in the 96% of the genomes analysed; RasI/R QS system is presented as red and is found in the 50% of the genome analysed and in particular not in RSSC species from the phylotype II.

RSSC strains possess at least four cell–cell signalling QS systems; as depicted in Figure 1 and mentioned above. Two of these, called SolI/R and RasI/R, are archetypical AHL QS systems consisting of a LuxI AHL synthase and a cognate LuxR‐family sensor/regulator. Another recently identified cell–cell signalling system uses anthranilic acid as a novel signal that is detected by the LysR family regulator RaaR sensor/receptor playing a role in virulence by regulating several loci (Song et al., 2020, 2022). The fourth and central system is the Phc QS system, which relies on a methyltransferase called PhcB that catalyses the formation of (*R*)‐3‐OH MAME or (*R*)‐3‐OH PAME signals (Flavier et al., 1997; Kai et al., 2015). A two‐component system consisting of PhcS‐PhcRQ is then involved in signal perception and transduction, resulting in the activation of the PhcA regulator (Clough et al., 1997; Kai, 2023; Takemura et al., 2021; Figure 1). PhcA then serves as the master regulator, controlling the expression of the majority of loci associated with virulence in RSSC. Knock‐out mutations in *phcA* lead to significant phenotypic changes, causing the bacteria to invade the host xylem in a low‐population state and resulting in a reduction of the expression of around 40 virulence factor loci compared to the wild type (Kai, 2023). Because of the central role of the Phc in regulating virulence in RSSC, this system has been extensively studied and has been very recently reviewed (Kai, 2023); this microreview focuses on the role of AHL QS systems in RSSC as well as their occurrence, organization and integration with the central Phc system.

## 3 THE WELL‐CONSERVED SolI/R AHL QS SYSTEM

*N*‐acyl‐homoserine lactone type signals (AHLs) are the most common QS cell–cell signals in *Proteobacteria* and are involved in regulating gene expression of many bacterial community phenotypes including pathogenicity (Papenfort & Bassler, 2016; Qin & Bassler, 2022). AHLs are diffusible molecules composed of a fatty acyl chain linked to a lactonized homoserine through an amide bond at the α position, where the lactone ring is unsubstituted in the β‐ and γ‐positions. The AHL signals are classified according to their acyl chains, which vary in their degree of saturation, oxidation, and substitution at the third carbon of the chain. The lengths of the acyl chains range from 4 to 20 carbons and their biosynthesis is performed by a LuxI‐family synthase and occurs most commonly via two substrates: *S*‐adenosylmethionine and fatty acid acyl–acyl carrier proteins. The AHLs are then detected by binding to a cognate LuxR‐family receptor leading most commonly to homodimerization, which then binds to specific DNA sequences called lux boxes, affecting target gene transcription. One of the targets is the *luxI* gene, thus generating a positive feedback loop. At low population densities, cells produce a basal level of AHLs; as the cell density increases, AHLs accumulate and reach a threshold (quorum) that allow binding to the cognate LuxR protein (Bassler, 2002; Fuqua & Greenberg, 2002). The LuxR‐family receptors/regulators consist of two modular domains: the N‐terminal domain binds to AHL, whereas its C‐terminal region forms a DNA binding helix–turn–helix motif. The binding of AHL to the N‐terminal domain allows the formation of the homodimers and stabilizes the LuxR protein. LuxI‐like proteins are highly conserved, whereas LuxR‐like proteins are more variable, with only 18%–25% similarity even among the same genus or species. Recent bioinformatic and metagenomic studies have shown that the majority of proteobacterial genomes harbour an excess of QS *luxR* family transcriptional regulators compared to QS *luxI* homologues; these *luxR*‐like genes are called *luxR* solos and they are more abundant and predominant than complete *luxI/R* QS systems (Bez et al., 2023).

In the RSSC, the SolI/R AHL QS system was initially identified in the *R. solanacearum* strain AW1 (Alberty et al., 1997); the *solR* and *solI*, which are *luxR* and *luxI* homologues respectively, are genetically linked and localized in the chromosome, showing a convergent arrangement (‘bidirectional’ genes head‐to‐head located on opposite strands and transcribed divergently). SolI is responsible for the biosynthesis of *N*‐hexanoyl‐l‐homoserine and *N*‐octanoyl‐l‐homoserine lactones (C6‐ and C8‐AHLs) and SolR responds to both types of AHLs (Figure 1).

Surprisingly, *solI*/*R* mutants do not result in a significant reduction of virulence; in fact this QS system controls the expression only of a small number of loci, most prominently it regulates *aidA* in the strain AW1 (Alberty et al., 1997). In RSSC strain UW551, the three genes *aidA*, *aidC*, and *lecM* (these are genetically adjacent to the *solI*/*R* locus) are positively regulated by the SolI/R AHL QS system (Meng et al., 2015). However, not all the RSSC strains possess the *aidA* and *aidC* genes, for example, the two genes are absent in the RSSC strain GMI1000. In the strain UW551, mutants lacking *aidA*, *aidC* or *lecM* result in a notable decrease in virulence on tomatoes at 20°C, with a less pronounced effect at 28°C. Furthermore, the survival capabilities of *lecM* and *aidC* mutants are reduced in potato tubers at the storage temperature of 4°C, as well as their biofilm formation ability.

Comparative genomic analysis of RSSC also supports that most strains of the RSSC are tropical and do not harbour the *aid* genes; it has therefore been hypothesized that the Aid proteins are involved in the adaptation and virulence in cold environments (Meng et al., 2015). Interestingly, AidA is also present in the human‐pathogenic strains belonging to the *Burkholderia cepacia* complex, where it is a virulence factor in the *Caenorhabditis elegans* model (Huber et al., 2004).

The expression of *solI*/*R* is significantly regulated by the Phc system creating a hierarchy closely linked to cell number/density; the Phc gene targets are regulated when cell density surpasses 10^7^ cells/mL whereas the SolI/R targets are switched on when densities reach 10^8^ cells/mL (see below; Flavier et al., 1997). The significance of this hierarchy is yet unknown and could be a way to provide timely adaptation/response to different cell densities. Hierarchical organization of multiple QS systems in bacteria is not novel and has been reported in other bacteria, for example the very well‐studied QS systems of *Pseudomonas aeruginosa* (Venturi, 2006).

## 4 THE RECENTLY IDENTIFIED RasI/R QS SYSTEM

Some strains of the RSSC possess an additional LuxI/R QS system designated as RasI/R (Yan et al., 2022). This system is located in the megaplasmid of some RSSC genomes and the two genes are organized in tandem arrangement (both genes *luxI* and *luxR* on the same strand). The primary AHL signal produced by RasI is the *N*‐(3‐hydroxydodecanoyl)‐homoserine lactone (3‐OH‐C12‐AHL); however, small quantities of *N*‐(3‐hydroxytetradecanoyl)‐homoserine lactone (3‐OH‐C14‐AHL) are also produced (Figure 1). The RasI/R QS system responds primarily to the signal 3‐OH‐C12‐AHL, followed by 3‐OH‐C14‐AHL and 3‐OH‐C10‐AHL.

RSSC strains of phylotype I, III and IV harbour RasI/R whereas strains of phylotype II do not; the RasI/R system of phylotype I and III are more closely related in their primary structure. Unlike what is observed for the SolI/R QS system, the RasI/R is involved in modulating virulence via the regulation of the virulence‐associated factors such as cellulase (CEL) production, motility, biofilm formation and the oxidative stress response (Yan et al., 2022). A transcriptome study has evidenced that RasI/R regulates the expression of over 154 loci including ABC transporters, transcriptional regulators, genes involved in stress resistance and detoxification, type III secretion systems, protein metabolism, flagellum synthesis, motility, chemotaxis, tricarboxylic acid cycle, fatty acid metabolism and small molecule metabolism (Yan et al., 2022). This is evidence that this QS system has evolved as an important regulator of virulence as well as being a more global regulator in some RSSC strains.

## 5 OCCURRENCE, HOMOLOGY AND GENETIC ORGANIZATION OF SolI/R AND RasI/R AHL‐QS SYSTEMS

**FIGURE 2:**
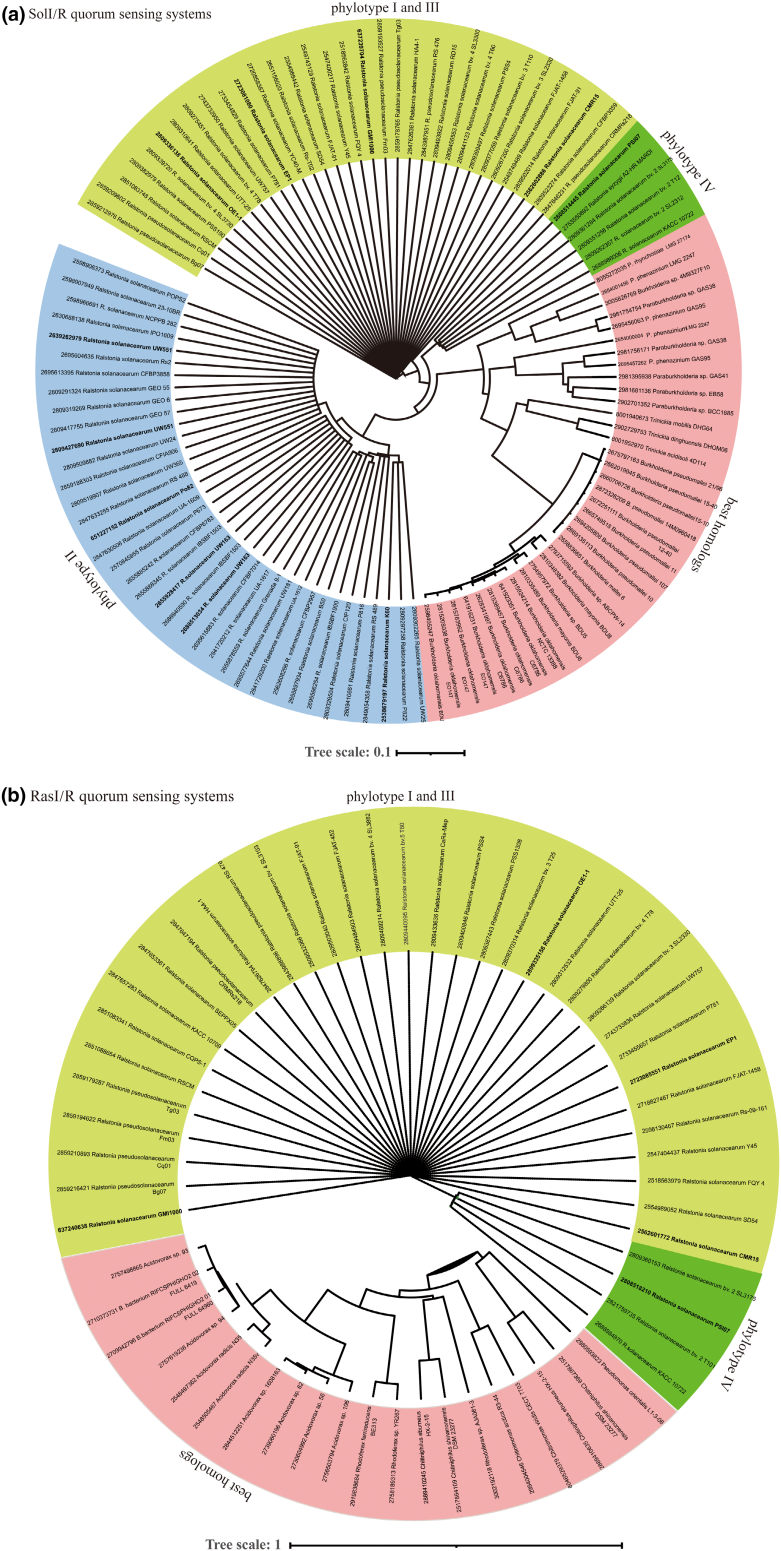
Taxonomic distribution and classification of SolI/R quorum‐sensing (QS) system and RasI/R QS system among proteobacterial genomes. Genomes of *Proteobacteria* from IMG database were searched for the presence of SolI/R and RasI/R genes using the ‘top homologue’ search feature in JGI IMG. A tree of the SolI/R (a) and RasI/R (b) representatives was made by aligning their protein sequences using Clustal Omega with standard settings (Sievers et al., 2011), and then using FastTree with the alignment as input with standard settings (Price et al., 2009). The tree is annotated at species level and the names of the best characterized and studied *Ralstonia solanacearum* species complex (RSSC) genomes are indicated in the tree in bold. In the external rings are clustered the RSSC genomes belonging to the four phylotypes. The best homologues hits belonging to bacterial genera that are not *Ralstonia* are grouped as ‘best homologues’. The tree is visualized and annotated using iTOL (Letunic & Bork, 2021).

In order to investigate the distribution and conservation of AHL‐QS LuxI/R systems among the RSSC strains, a systematic bioinformatic analysis has been performed here. A total of 127 RSSC genome sequences deposited in the Genome Institute Integrated Microbial Genomes (JGI IMG) repository were sourced. All potential AHL‐QS LuxRs identified contained the typical two signature Pfam domains (PF03472 autoind_bind domain at N‐terminal and PF00196 DNA‐binding HTH domain at C‐terminal) and all potential AHL‐QS LuxIs contained the autoinducer synthase Pfam domain (PF00765). Out of 127 RRSC genomes analysed, 122 genomes (96%) possess SolI/R, while 61 (50%) harboured the RasI/R system. In approximately 3.9% of genomes, we did not detect either a SolI/R or RasI/R system homologues. By using the JGI IMG ‘top homologue’ search feature, we found that the SolI/R system is very conserved among the RSSC with 98%–85% of amino acid (aa) identity (Figure 2a). On the other hand, outside of the RSSC, the best SolI/R homologues were found in *Trinickia* spp. (about 71% aa identity or higher), and *Burkholderia* spp. (about 67%). Within the *Burkholderia* spp. group, the most extensively characterized SolI/R‐homologue system is the BpsI/R system identified in *B. pseudomallei*. This system also synthesizes C8‐HSL as the primary lactone and plays a crucial role in the formation of biofilms and movement (Gamage et al., 2011). As evidenced by the phylogenetic tree (Figure 2a), and based on their primary sequence homology, SolI/R systems clearly clades according to the type of phylotype. In particular, SolI/R of the phylotype I and III were closely related and distinct from SolI/R of phylotype II.

The RasI/R system was found in a smaller number of RSSC strains (Figure 2b), being present only in the half of the genomes sequenced, but it shares a very high amino acid identity (100%–89%) among the members of the RSSC. Interestingly, the RasI/R system has a high aa identity (about 50%) with a LuxI/R‐like system present in several bacterial genera such as *Acidovorax*, *Burkholderia*, *Chitinomonas*, *Chitiniphilus*, and *Rhodoferax*. However, the best homologues for RasR were found in *Trinickia* spp. and *Variovorax* spp. (58% aa identity), while the best homologues for RasI, in its primary structure, was found to be MbaI (52% aa identity), which is part of the MbaI/R QS signal producing 3‐OH‐C10‐AHL and 3‐OH‐C12‐AHL in *Methylobacter tundripaludum* (Puri et al., 2017). This result possibly indicates that RasR and RasI originated from distinct sources and were independently acquired at a later stage.

This analysis revealed that the SolI/R system is widely distributed among the RSSC members, and it is also conserved with other bacterial genera. On the other hand, RasI/R is less distributed among the RSSC members and shares very high identity.

## 6 HIERARCHICAL QS MODULATION AND THE POSSIBLE ROLE OF OTHER SIGNALS

RSSC pathogenesis is regulated by an elaborate, hierarchical network of regulators that respond to environmental stimuli and cell–cell signals (Figure 1). Virulence loci such as the EPS that obstructs the xylem vessels causing the wilt symptom, the production of putrescine that accelerates wilt disease (Lowe‐Power et al., 2018), two cellulolytic enzymes (Egl and CbhA; Liu et al., 2005), and motility are all modulated by the Phc system in response to different cues and stimuli (Meng et al., 2011; Tans‐Kersten et al., 2001; Yao & Allen, 2006, 2007). Regulation of EPS production is also connected with lectin and β‐1,4‐cellobiohydrolase enzyme activities (Hayashi et al., 2019; Senuma et al., 2023). LecM, EPS I and β‐1,4‐cellobiohydrolase are involved and interconnected with the modulation of the QS signalling pathway intimately linking virulence factors to cell–cell signalling. The mechanisms governing these two‐way controls are currently unknown and unravelling them represents an important future challenge.

As mentioned above, the Phc QS system positively regulates the RasI/R and SolI/R QS systems via the PhcA master‐regulator; it needs to be established whether this transcriptional regulation is direct or indirect (Perrier et al., 2018; Takemura et al., 2021). SolI/R is also under regulation of the RasI/R system, thus placing SolI/R at the bottom of the hierarchical QS response. This Phc, RsaI/R and SolI/R hierarchy will most likely result in the timing of their target gene expression possibly allowing RSSC strains to be best adapted to the multiple plant‐associated environmental conditions.

It is also worth noting that some secondary metabolites and other types of secreted molecules are increasingly recognized as intra‐ and interspecies cell–cell signalling molecules (Han et al., 2017; Tarkka et al., 2009). These include ralsolamycin (synonym ralstonin A; Baldeweg et al., 2017; Murai et al., 2017; Spraker et al., 2016), ralfuranone (Wackler et al., 2011), the yersinabactin‐like siderophore (Kreutzer et al., 2011) and micacocidin (Kobayashi et al., 2000). Interestingly, some secondary metabolites are modulated by the Phc QS such as the ralsolamycin/ralstonins (Li et al., 2017; Li et al., 2022), ralfuranone (Kai et al., 2014), and the siderophore staphyloferrin B (Bhatt & Denny, 2004). Recently, anthranilic acid has been reported to play dual roles in intraspecies and interkingdom signalling (Song et al., 2020), and the sensor/receptor protein RaaR, which responds to anthranilic acid, modulates the expression of the *phc* and *solI/R* systems (Song et al., 2022).

In summary, RSSC strains use a complex set of hierarchical cell–cell communication systems to coordinate expression of virulence factors for pathogenesis as well as other secondary metabolites. Having multiple QS systems offers several advantages for the bacterial lifestyle, such as (i) the ability to diversify the communication by responding to various environmental cues and coordinating different types of behaviours; (ii) adaptation to varied environments by being able to respond to specific environmental factors or stresses by modulating the behaviours based on the presence of particular signalling molecules; (iii) provision of redundancy and robustness, so if one system is compromised or fails then other systems can still function, maintaining the ability to coordinate population‐wide activities; (iv) contributing to population heterogeneity, where subpopulations of bacteria respond differently to various signals and to specific environmental challenges; (v) evolutionary flexibility allowing bacteria to adapt and evolve over time and (vi) allowing for fine‐tuned regulation of gene expression and behaviour.

## 7 CONCLUSIONS AND FUTURE PERSPECTIVES

The RSSC strains possess a complex contact‐independent cell–cell signalling response integrating four QS systems, namely the anthranilic acid QS system, Phc, RsaI/R, and SolI/R QS systems. The Phc QS system holds a central role, governing the RasI/R and SolI/R QS systems, and being regulated in turn by the novel anthranilic acid system. All of these QS systems are essential for full virulence gene expression; it is likely that RSSC strains sense and respond to a variety of environmental and/or host signals affecting specific QS system(s). The roles of two AHL QS systems in RSSC are gaining interest and importance. The involvement of the RasI/R QS system in virulence has been only documented in strain EP1 thus far. Consequently, given the evolutionary diversity within the RSSC, exploring whether different strains exhibit distinct roles and responses to this AHL QS system is necessary. In contrast to RasI/R, the SolI/R system is highly conserved in the RSSC. In strains AW1 and UW551, the SolI/R QS system targets *lecM*, *aidA*, and *aidC* loci, which are associated with virulence, particularly at 20°C. Future transcriptome studies in both laboratory culture media and in planta settings will provide more insights into the regulon governed by SolI/R.

In order to determine the precise role and importance of AHL QS systems in the RSSC further experimentation is still needed. The ubiquitous presence of SolI/R and the spread and evolution of RasI/R highlight the importance in the biology of contact‐independent cell–cell signalling via AHLs in this very important group of phytopathogenic bacteria.

## CONFLICT OF INTEREST STATEMENT

The authors declare that they have no conflicts of interest.

## Data Availability

Data sharing in not applicable to this article as no new data were created or analysed.
